# Impact of sella floor reconstruction on Rathke Cleft Cyst recurrence: a systematic review and meta-analysis

**DOI:** 10.1007/s11102-025-01521-4

**Published:** 2025-04-14

**Authors:** Ryan Beerling Dolovac, James King, Christopher Ovenden, Jeremy Kam, Yi Yuen Wang, Tony Goldschlager, Mendel Castle-Kirszbaum

**Affiliations:** 1 School of Medicine, University of Western Australia, Perth, Australia; 2 Department of Neurosurgery, Royal Melbourne Hospital, Melbourne, Australia; 3 Department of Surgery, University of Melbourne, Melbourne, Australia; 4 Department of Neurosurgery, Royal Adelaide Hospital, Adelaide, Australia; 5 Faculty of Health and Medical Sciences, Adelaide Medical School, Adelaide, Australia; 6 Department of Neurosurgery, Monash Health, Melbourne, Australia; 7 Department of Surgery, Monash University, Melbourne, Australia; 8 Department of Neurosurgery, St Vincent’s Health, Melbourne, Australia; 9 Department of Surgery, University of Melbourne, Melbourne, Australia

**Keywords:** Rathke, Cyst, Pituitary, Recurrence, Reconstruction, Flap

## Abstract

**Background:**

The optimal surgical technique for managing Rathke’s Cleft Cyst (RCC) remains unclear. Leaving the sellar defect open (marsupialisation) after transsphenoidal surgery facilitates ongoing drainage of cyst contents, but cannot be performed in the setting of an intraoperative cerebrospinal fluid (CSF) leak. The effects of intraoperative CSF leaks and sellar floor reconstruction on RCC recurrence require further investigation.

**Methods:**

A systematic literature search was conducted for studies reporting RCC recurrence following transsphenoidal surgery, with data on intraoperative CSF leak rates and skull base reconstruction. Studies were classified based on surgical technique: cyst wall resection vs. fenestration, and open (no reconstruction) vs. closed (reconstructed) sellar floor.

**Results:**

Nineteen studies, comprising 1,076 patients, were included. The overall radiological RCC recurrence rate was 19.8% over a mean follow-up of 50.4 months. The recurrence rate in closed sella surgeries was significantly higher (32.1%) than in open sellar cases (14.0%) (OR 2.28, 95% CI: 1.41–3.67, *p* < 0.05). Intraoperative CSF leak occurred in 29.1% of cases. Patients with CSF leaks had a higher recurrence rate (23.4% vs. 12.9%), though meta-analysis demonstrated only a non-significant trend (OR 1.67, 95% CI: 0.95–2.96). Subgroup analysis revealed that intraoperative CSF leaks were significantly associated with increased recurrence after fenestration (38.5% vs. 18.4%, *p* = 0.03), and cyst wall resection (21.7% vs. 7.8%, *p* = 0.004). In the setting of an intraoperative CSF leak, there was a trend for lower recurrence when cyst wall resection was attempted (21.7% vs. 38.5%, *p* = 0.09).

**Conclusion:**

Patients undergoing transsphenoidal surgery for RCC experience high rates of postoperative radiological recurrence. Cyst fenestration while maintaining an open sellar floor (marsupialisation into the sphenoid sinus) is associated with a significantly lower risk of recurrence at over 4 years follow-up. Intraoperative CSF leaks were less strongly associated with cyst recurrence, suggesting that watertight reconstruction, rather than the leak itself, is the primary driver of reaccumulation. When a closed sella is necessitated by intraoperative CSF leak, the addition of cyst wall resection may be associated with a lower rate of recurrence than fenestration alone but must be weighed against a higher risk of AVP-deficiency.

**Supplementary Information:**

The online version contains supplementary material available at 10.1007/s11102-025-01521-4.

## Introduction

Rathke Cleft Cysts (RCC) are endodermal cysts interposed between the anterior and intermediate lobes of the pituitary gland formed due to incomplete involution of Rathke’s pouch [1]. RCC may become symptomatic due to mass effect on parasellar structures, leading to hypopituitarism, visual loss, headache, and reduced quality of life [2, 3]. Surgical techniques to address mass effect include cyst aspiration, fenestration, and cyst resection, however recurrence remains a significant risk. Fenestration and drainage is often preferred, as although cyst wall resection has been associated with lower recurrence rates, it is also poses a greater risk of postoperative endocrinopathy [1].

Leaving the sella floor open after transsphenoidal fenestration facilitates ongoing drainage (marsupialisation) of the cyst into the sphenoid sinus, which may prevent recurrence. However, if an intraoperative cerebrospinal fluid (CSF) leak is encountered, the sella floor must be reconstructed to prevent a postoperative leak and its sequelae. It is not clear if intraoperative CSF leaks or sella floor reconstruction predispose to cyst reaccumulation compared to marsupialisation. Here we present a systematic review and meta-analysis to elucidate the effect of sella floor reconstruction and intraoperative CSF leak on recurrence rates in RCC to guide surgical decision making.

## Methods

A systematic search of the literature was conducted using the PubMed, Embase, Cochrane Library, and Scopus databases in accordance with the Preferred Reporting Items for Systematic Reviews and Meta-Analyses (PRISMA) statement [4]. All studies from database inception until November 2024 were queried using the search string:

### Rathke or rathke’s

Exclusion criteria were single case reports, studies published in languages other than English, and studies where data specific to operative technique and recurrence rate could not be extracted. The references of included studies were also hand-searched to identify additional eligible studies.

After title and abstract review, full-text review was performed in appropriate studies to determine suitability for inclusion. Inclusion criteria were defined as: (1) Trials, prospective and retrospective cohort studies that report recurrence outcomes of transsphenoidal (endoscopic or microscopic) surgery on RCC; (2) Clear description of operative technique; (3) Reports intraoperative CSF leak rate.

Included studies underwent blinded, independent data extraction by two reviewers including study year, study size, surgical technique, intraoperative CSF leak rate, and recurrence rate.

Classification of surgical technique was performed independently. Given a lack of consistent nomenclature in the literature, a classification scheme for surgical techniques to RCC was defined a priori. Resection was defined as subtotal or total removal of the cyst wall, while fenestration was defined as a smaller opening in the cyst wall to facilitate drainage of the cyst contents. Cysts could be fenestrated from below into the sphenoid sinus, or above into the suprasellar cisterns; if this pathway was left open to drain, it was termed marsupialisation. If a watertight sella floor reconstruction was attempted, this was termed a closed sella, while if no reconstruction was attempted (facilitating marsupialisation into the sphenoid sinus), it was termed an open sella. Multilayer reconstruction with mucosa, fascia lata, or allograft, was considered a closed sella. Thus, surgical technique could be classified into: Cyst wall resection with open sella; cyst wall resection with closed sella; fenestration into sphenoid sinus with an open sella (sphenoid marsupialisation); fenestration into sphenoid sinus with a closed sella (cyst drainage); and fenestration into suprasellar cisterns with a closed sella (suprasellar marsupialisation).

The association between open and closed sella surgical techniques, and intraoperative CSF leaks to cyst recurrence rates was analysed, and meta-analysis with the common effects model was performed. Interstudy heterogeneity was measured with the Cochran’s Q test. An alpha < 0.05 was defined as statistically significant. Analysis was performed using R 4.4.2.

## Results

A total of 19 studies were identified from the systematic literature search (Supplementary Fig. 1), comprising 1076 patients (Table 1) [5–23]. All were retrospective cohort studies. Where data were available, the patient population demonstrated a female predominance (67.0%, 682/1018), and a mean age of 43 years. Mean RCC size diameter was 16.77 mm (*n* = 527). Presenting symptoms included headache (59.6%, 438/735), vision loss (48.3%, 281/582), and endocrinopathy (37.7%, 125/332). The overall rate of radiological RCC recurrence was 19.8% (213/1076) after a mean follow-up of 50.4 months. Of these, 51.6% (110/213) were reoperated on. Postoperative CSF leaks (3.2%, 29/918) and meningitis (1.9%, 14/756) were uncommon complications.

**Table 1:** Summary of studies analysing RCC recurrence

Author (year)	*N*	Surgical strategy	F/U (months)	Recurrence* rate (%)	Reoperation^ rate (%)	Postop Permanent DI (%)	Postop Hypopituitarism (%)
Aho (2005)	118	Cyst wall Resection	NR	17.8	10.2	18.6	1.7
Algattas (2024)	148	(Fenestration/ Marsupialisation) (88) + Cyst wall resection (60)	40	14.9	8.8	4.7	3.4
Arko (2021)	31	Fenestration/ Marsupialisation	28	25.8	0	NR	NR
Benveniste (2004)	62	Fenestration (56) + Cyst wall resection (6)	28	16.1	12.9	NR	NR
Eide (2022)	21	Fenestration	38	28.6	28.6	NR	NR
Kinoshita (2016)	91	Fenestration/ Marsupialisation	80	39.6	14.3	NR	NR
Limb (2021)	23	NR	96	47.8	39.1	0.0	0.0
Lin (2019)	109	(Fenestration + Marsupialization) (94) + Cyst wall resection (15)	67	26.6	9.2	NR	NR
Frank (2005)	22	Marsupialization (16) + Cyst wall resection (6)	33	4.5	4.5	4.6	NR
Jiang (2018)	13	Cyst wall resection	17	0	0	0.0	0.0
Kasperbauer (2002)	29	Mixed (NR)	79	27.6	10.3	NR	NR
Kim (2004)	53	Fenestration (17) + Cyst wall resection (36)	31	11.3	11.3	NR	NR
Koutourousiou (2009)	14	Cyst Wall Resection	29	14.3	0	7.1	NR
Lillehei (2011)	82	Cyst Wall Resection (18) + Fenestration (64)	46	10.7	7.3	0.0	NR
Madhok (2010)	35	Fenestration/ Marsupialisation	19	5.7	0	0.0	0.0
Nakase (2024)	35	Fenestration/ Marsupialisation	94.7	42.8	14.3	5.7	8.6
Wedemeyer (2019)	91	Cyst Wall Resection	69	21.8	19.8	4.4	NR
Yamada (2022)	27	Fenestration/ Marsupialisation	52	29.6	0	3.7	0.0
Zhang (2021)	72	Cyst wall Resection	61	0	0	NR	4.2

*= Recurrence was based on radiological recurrence as assessed with MRI in all studies

^ = Reoperation for recurrence (not counting repeat reoperations)

### Open vs. closed sella

**Fig. 1:**
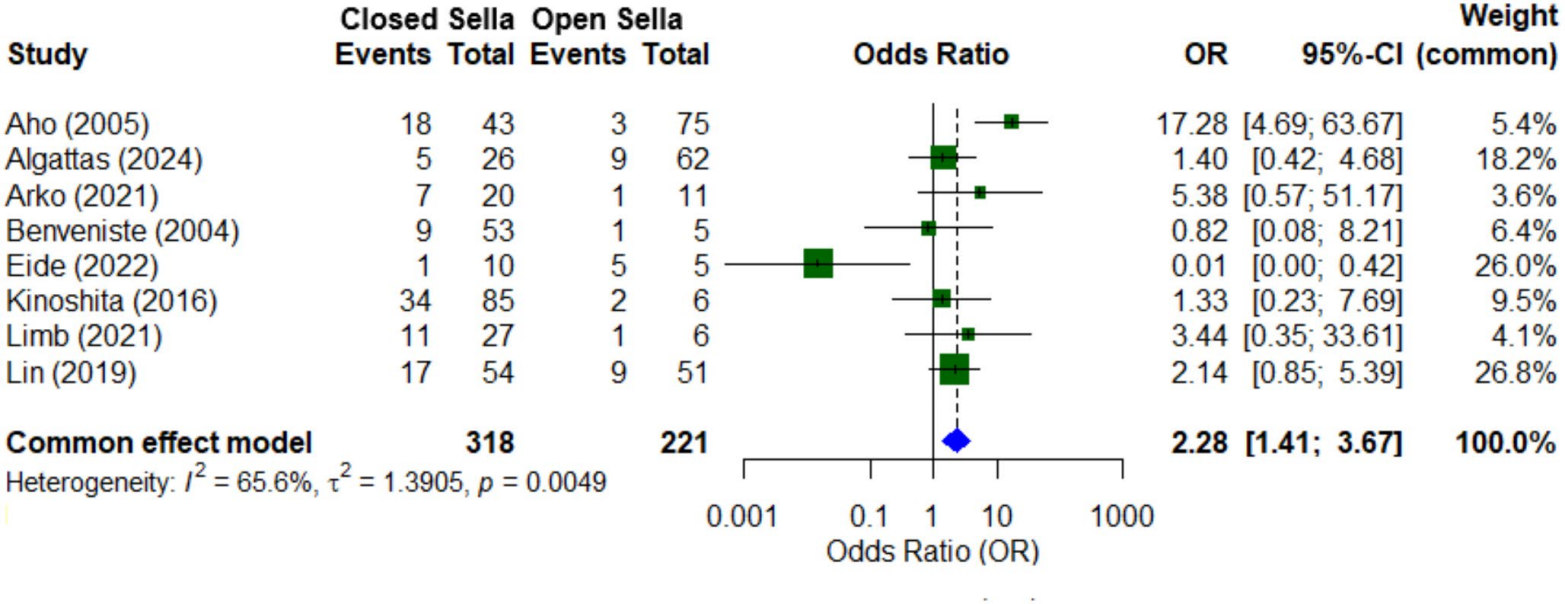
Forrest plot depicting meta-analysis of radiological recurrence in open vs. closed sella

Sella floor reconstruction data were available in eight studies totalling 539 patients [5–12]. The recurrence rate across studies ranged from 12.5 to 48%, with a mean follow-up of 53.9 months. The recurrence rate in closed sella surgeries (32.1%, 102/318) was greater than that of cases with an open sella (14.0%, 31/221). Meta-analysis demonstrated closed sella cases have more than twice the odds of recurrence compared to open sella cases (2.28 (95% CI: 1.41–3.67)) (Fig. 1). Significant heterogeneity was observed between studies (I² = 65.6%), while Egger’s test (*p* = 0.36) indicated no statistically significant evidence of publication bias. Only 2 small studies reported reoperation rates by sella reconstruction technique, with no significant difference seen in either study [7, 9].

The effect of cyst wall resection on recurrence and hormonal outcomes was also analysed. There was a significant difference in recurrence rate between patients undergoing fenestration vs. cyst wall resection with an open sella defect (*n* = 159, 20.2% vs. 4%, *p* = 0.001), but not in the closed sella cohort (*n* = 184, 33.3% vs. 41.9%, *p* = 0.31). Postoperative AVP-deficiency (DI) occurred over 3-fold as often after cyst wall resection compared with fenestration or marsupialization in the study database (*n* = 489, 10.6% vs. 3.2%, *p* = 0.003). By contrast, there was no difference in the rates of postoperative hypopituitarism between surgical techniques (*n* = 415, 3.7% vs. 2.8%, *p* = 0.58).

### Intraoperative CSF leak

**Fig. 2:**
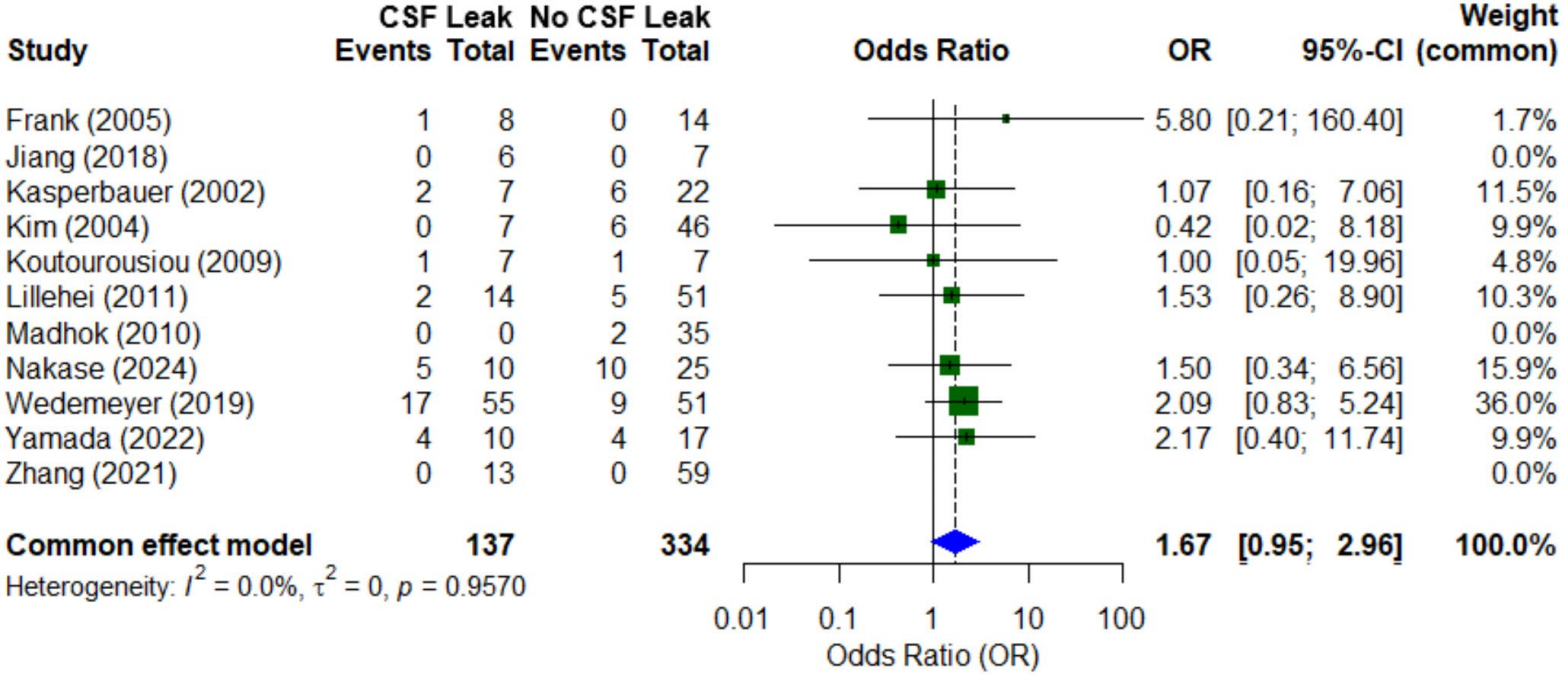
Forrest plot depicting meta-analysis of radiological recurrence by intraoperative CSF leak status

A total of eleven studies were included in the analysis, comprising 473 patients, with intraoperative CSF leak data available from 471 operations [13–23]. The pooled CSF leak rate was 29.1% (137/471). The recurrence rate across studies ranged from 0 to 43%, with a mean follow up of 54 months. In patients with an intraoperative CSF leak, the recurrence rate was 23.4% (32/137), while in patients without a leak, the rate was 12.9% (43/334). Meta-analysis demonstrated a non-significant trend toward greater recurrence with intraoperative CSF leak (OR 1.67 95% CI: 0.95–2.96) (Fig. 2). Heterogeneity between studies was minimal (I² = 0.0%), and Egger’s test (*p* = 0.37) suggest no publication bias. On simple pooled analysis however, the association of intraoperative CSF leak to recurrence was significant (*p* < 0.001), likely because studies without recurrence events could be included in the analysis.

Recurrences requiring reoperation occurred in 8.2% (39/473) of cases, reported across six studies [13, 15, 16, 18, 20, 21]. The rate of reoperation was similar in patients with (1.9%, 1/51) and without (3.1%, 6/185) intraoperative CSF leaks (*p* > 0.99).

Subgroup analysis was performed for specific surgical techniques. In patients undergoing cyst fenestration alone, there was a significant difference in radiological recurrence rates between patients who experienced an intraoperative CSF leak (38.5%) vs. no intraoperative CSF leak (18.4%) (*n* = 113, *p* = 0.03). In patients undergoing cyst wall resection, there was a significant difference in recurrence rates between patients who experienced an intraoperative CSF leak vs. no intraoperative CSF leak (*n* = 211), (21.7% with CSF leak vs. 7.8% without, *p* = 0.004).

In patients with an intraoperative CSF leak, there was a non-significant trend towards lower risk of recurrence with cyst wall resection compared with fenestration or marsupialization alone (*n* = 109) (38.5% vs. 21.7%, *p* = 0.09). In contrast, this trend was statistically significant in patients without CSF leaks (*n* = 215) (18.4% vs. 7.8%, *p* = 0.02).

## Discussion

Patients undergoing transsphenoidal surgery for RCC experience high rates of postoperative recurrence. Maintaining an open sella floor (cyst marsupialisation) is associated with a significantly lower risk of cyst recurrence at over 4 years follow-up compared to when the sella floor is surgically reconstructed. Intraoperative CSF leaks were less strongly associated with cyst recurrence, suggesting that watertight reconstruction, rather than the leak itself, is the driver of reaccumulation. When a closed sella is necessitated by intraoperative CSF leak, the addition of cyst wall resection may be associated with a lower rate of recurrence than fenestration alone.

Recurrence of RCC is a common problem, occurring in one-fifth of the cohort. Cyst wall resection, marsupialisation [24], and instillation with sclerosants [5, 8, 18] have all been trialled to prevent recurrence, with varying degrees of success. Maintaining an outflow pathway for cyst contents to drain (marsupialisation) would theoretically prevent reaccumulation, so long as the outflow tract remains patent. Simple marsupialisation involving infrasella fenestration of the RCC and no sella floor reconstruction requires no foreign materials or mucosal harvesting, and appears to reduce the rate of recurrence. However, cicatricial stenosis or obliteration of the outflow tract is not uncommon. Techniques to preserve the marsupialisation stoma include stenting, mucosal coupling [25], and mucosal grafting. Nonabsorbable (tympanostomy tube, sialastic tube) [26, 27] and bioabsorbable steroid-eluting [28] stents provide a physical scaffold to maintain patency [29] and have demonstrated good efficacy in small case series. Invagination of the sphenoid mucosa, free mucosal graft, or a nasoseptal flap into the cyst cavity obliterates the potential space, and encourages continuity with the sphenoid sinus by forming an epithelialised tract [24, 27]. No study directly compares these adjuncts to simple marsupialisation for prevention of recurrence.

By virtue of its embryological origins, RCC after often intimately adherent to the anterior pituitary and stalk. Consequently, complete resection of the cyst wall is associated with a significant risk of AVP-deficiency (diabetes insipidus) [5, 16, 23, 30, 31] and anterior pituitary dysfunction [8, 23, 30, 31]. Cyst wall resection has been shown to reduce the chance of recurrence in RCC [32], but must be balanced against the risk of new endocrinopathy. For patients with an intraoperative CSF leak precluding marsupialisation, the addition of cyst wall resection may mitigate the increased risk of recurrence associated with a closed sella. In this review, the absolute difference in recurrence rates was substantial (38.5% vs. 21.7%) and approached statistical significance (*p* = 0.09). This must be weighed against the risk of iatrogenic endocrinopathy, particularly AVP-deficiency. Postoperative AVP-deficiency (DI) occurred over 3-fold as often after cyst wall resection compared with fenestration or marsupialization. By contrast, there was no difference in the rates of postoperative hypopituitarism between surgical techniques. Patients with preoperative hypopituitarism from RCC are unlikely to recover normal function, particularly if there is panhypopituitarism or AVP-deficiency [5, 6]. Patients with recurrent RCC, preoperative endocrinopathy (particularly AVP-deficiency), a clear plane of separation between the cyst wall and stalk, or in whom recurrent surgeries may not be advisable, may be candidates for cyst wall resection in the event of an arachnoid breach.

In suprasellar cysts, an alternative pathway for marsupialisation is into the suprasellar subarachnoid cisterns. The longevity of suprasellar and sphenoid marsupialisation tracts appear similar, although data is limited [10]. Suprasellar marsupialisation can be achieved through a suprasellar transsphenoidal transtubercular approach or a craniotomy with subfrontal dissection. The transsphenoidal trajectory provides an en face view of the cyst and suprasellar space, but the associated subarachnoid dissection introduces the morbidity of vascularised flap reconstruction [33]. The location and morphology of the optic chiasm should be assessed preoperatively, as a prefixed chiasm or narrow infrachiasmatic window may limit access through a transtubercular approach [34].

**Fig. 3:**
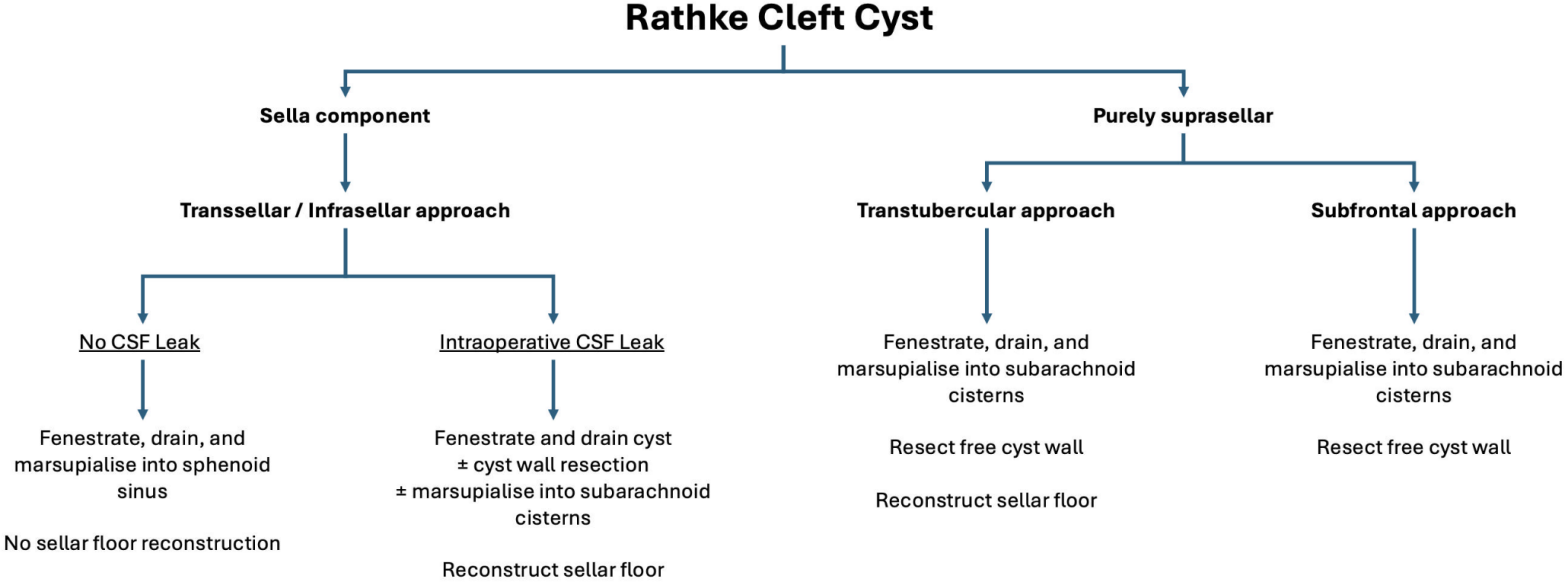
A proposed algorithm for RCC management

Based on the available data, we propose a management algorithm for RCC (Fig. 3). Cysts predominantly located within the sella are ideally fenestrated from below using a transsphenoidal transsellar/infrasellar approach. Marsupialisation into the sphenoid sinus more than halves the recurrence risk (0.44 (95% CI: 0.28–0.71)) and can be augmented with stenting or mucosal inlay in recurrent cases. In cases with an intraoperative CSF leak, sella floor reconstruction is necessary, but tends to increase recurrence rates. The choice of reconstruction technique varies by CSF leak grade [35] and institution; we favour a graded approach [36]. Intraoperative conversion to cyst wall resection may lower the recurrence risk, however, this must be balanced with the increased risk of AVP-deficiency. Alternatively, the cyst can be marsupialised into the subarachnoid cisterns prior to sella floor reconstruction. For purely suprasellar cysts, marsupialisation into the subarachnoid space can be achieved from both above (craniotomy) or below (transtuberculum).

Our study has several limitations. All studies were retrospective and the majority had small sample sizes. There was significant heterogeneity in surgical technique, even when stratified by reconstruction technique, with variable use of vascularised mucosal grafts and flaps, fat, allograft, and fibrin-based adhesives. Intraoperative CSF leak and sella reconstruction are intimately tied, but were often not reported simultaneously, rendering them inseparable and limiting the number of datapoints available. Despite recurrence being a lifelong risk for patients with RCC, follow-up was relatively short (mean 50 months) and observed differences can not be extrapolated further. Recurrence was universally reported as radiological reaccumulation, which may not be clinically significant; only half of radiological recurrences require re-operation. Rates of meningitis, postoperative CSF leak, and reoperation were not reported stratified by sella reconstruction or intraoperative CSF leak, surgical technique (endoscopic vs. microscopic), or reoperation status, limiting comparisons. Studies without a recurrence event could not be included for common effects meta-analysis, limiting sample size and leading to discrepancies with pooled analysis. Finally, there was significant statistical heterogeneity during meta-analysis for intraoperative CSF leaks, limiting the generalisability of results.

## Conclusion

Recurrence is a common complication after surgery from RCC. When feasible, a sellar/infrasellar approach with fenestration and marsupialisation of the cyst into the sphenoid sinus without sella floor reconstruction is preferred and is associated with a lower rate of radiological recurrence than with sella reconstruction. In cases of intraoperative CSF leak, partial cyst wall resection can be attempted to mitigate the increased risk of recurrence associated with sellar reconstruction, but this must be balanced with the higher risk of AVP-deficiency. Prospective, randomised trials are required to confirm the optimal surgical strategy for RCC.

## Electronic supplementary material

Below is the link to the electronic supplementary material.

**Supplementary Material 1**: **Supplementary Fig. 1** – PRISMA flow diagram for systematic literature search.

## Data Availability

No datasets were generated or analysed during the current study.

## Code availability

N/A.
